# Supplementary material for: Modular Combinatorial DNA Assembly of Group B Streptococcus Capsular Polysaccharide Biosynthesis Pathways to Expediate the Production of Novel Glycoconjugate Vaccines
Source: Vaccines (Basel). 2025 Mar 6;13(3):279. doi: 10.3390/vaccines13030279 (PMC11946114; doi:10.3390/vaccines13030279)
Supplement: Supplementary file 1 [file vaccines-13-00279-s001.zip › vaccines-3411837-supplementary.pdf]

Supplementary file S1.

DNA sequences used for expression of GBS serotypes III, IV and V. The native start and stop codons have been removed, the prefix and suffix sequences highlighted in bold are added to allow sequences to be used in Start-Stop Assembly. Sequences from GBS-III were taken from accession number AL7668489, sequences from GBS-IV were taken from accession number AF355776, sequences from GBS-V were taken from accession number AF349539.

<u>GBS-III</u>

cpsE

AAGGGGTTGGTCTCATGTGGCTCTTCGATGAAAGAGAAGGAGAATATTCAAAAGATCATTATCGCCATGATTCA
GACTGTTGTAGTCTACTTCAGTGCAAGTCTGACTCTGACGCTGATTACCCCCAATTTTAAGTCAAACAAGGACTTA
TTGTTCGTTTTGCTGATCCATTATATTGTTTTCTATCTGAGTGATTTCTATCGTGACTTCTGGTCGCGCGGCTATCTT
GAGGAATTCAAGATGGTACTGAAATACTCGTTTTATTACATTTTTATCAGCTCATCCCTTTTCTTTATCTTCAAAAAC
TCCTTCACGACGACGCGCTTATCGTTTTTCACATTTATTGCTATGAATTCTATCTTGCTTTATTTATTAAACTCGTTTT
TAAAATATTATCGCAAGTACTCATACGCGAAGTTTTCTCGCGACACGAAAGTCGTATTGATTACCAATAAGGACAG
CCTGAGTAAGATGACGTTCCGCAACAAGTATGATCACAACTATATCGCTGTATGTATTTTAGATAGTAGCGAAAAA
GACTGCTACGACCTGAAACATAATTCATTGCGCATCATTAATAAGGATGCCCTGACTAGCGAATTAACATGTTTGA
CCGTGGACCAGGCTTTCATTAATATTCCAATTGAGTTGTTCGGGAAGTATCAAATTCAAGATATCATCAACGATATC
GAAGCCATGGGTGTTATCGTAAACGTGAACGTTGAGGCACTGAGTTTTGATAACATCGGTGAGAAACGTATTCA
GACTTTTGAGGGTTATTCGGTCATCACCTATAGCATGAAGTTTTACAAGTATAGTCACTTAATTGCGAAACGCTTT
TTAGACATCACAGGGGCTATTATCGGCCTTCTGATCTGTGGAATCGTAGCAATTTTCTTGGTGCCTCAGATCCGCA
AGGACGGTGGACCAGCCATTTTCTCACAGAATCGCGTAGGTCGCAATGGCCGTATTTTCCGTTTCTACAAGTTCC
GTTCTATGCGTGTAGATGCCGAGCAGATTAAAAAAGACTTGTTGGTGCATAACCAAATGACTGGACTGATGTTCA
AATTGGAGGATGACCCCCGCATCACCAAGATCGGGAAGTTCATCCGCAAAACTTCCATTGACGAACTTCCACAAT
TCTATAACGTCCTTAAAGGAGACATGTCTTTAGTCGGAACACGCCCGCCTACAGTAGACGAATATGAGAAGTACA
ACTCGACGCAAAAACGTCGTTTAAGTTTCAAGCCCGGGATTACAGGCCTGTGGCAAATCTCTGGGCGTAATAATA
TTACCGATTTCGATGAAATTGTCAAGTTGGACGTGCAATACATCAATGAATGGTCCATCTGGTCAGACATTAAGAT
TATTCTTCTGACGTTAAAGGTAGTATTGCTTGGAACTGGTGCAAAA**TAATGAAGAGCGACCAGAGACCCAACA**
CTG

cpsF

AAGGGGTTGGTCTCATGTGGCTCTTCGATGAAGATTTGTCTGGTCGGCTCATCAGGTGGTCATCTGGCCCATCT
TAATCTGCTTAAACCGATTTGGGAAAAAGAGGACCGTTTTTGGGTTACGTTTGACAAAGAAGATGCGCGCAGCA
TTTTGCGTGAAGAGATTGTGTACCACTGTTTTTTCCCAACAAACCGCAACGTAAAAAACCTTGTCAAAAACACCA
TTTTAGCGTTTAAGGTTTTGCGTAAGGAACGCCCTGATGTCATTATCAGCAGTGGCGCGGCAGTAGCGGTCCCG
TTTTTCTACATTGGTAAGTTATTCGGTTGTAAGACAGTTTATATCGAGGTATTCGACCGCATCGACAAACCTACGTT
GACCGGCAAATTAGTGTATCCAGTTACTGACAAGTTCATCGTACAATGGGAGGAGATGAAGAAGGTTTACCCTA
AGGCGATCAACTTAGGAGGAATTTTC**TAATGAAGAGCGACCAGAGACCCAACACTG**

cpsG

AAGGGGTTGGTCTCATGTGGCTCTTCGATGATCTTCGTAACGGTTGGGACTCACGAACAGCAGTTCAACCGTTT
GATTAAAGAAGTTGATCGTCTGAAAGGAACAGGAGCAATCGATCAGGAGGTtTTCATCCAAACAGGGTACAGC
GACTTTGAGCCCCAAAACTGCCAATGGTCAAAGTTCCTGAGCTATGACGACATGAACAGTTACATGAAGGAGGC
TGAAATTGTAATCACACACGGAGGTCCCGCAACATTTATGTCAGTTATCAGTTTGGGTAAATTGCCAGTCGTAGTG
CCGCGCCGTAAACAGTTCGGAGAACATATTAACGATCATCAGATCCAGTTTTTTAAGAAAATCGCCCATCTTTACC
CGCTTGCCTGGATTGAGGACGTGGATGGGTTGGCTGAAGTCCTTAAACGTAACATCGCCACTGAAAAATACCAG

GGCAACAACGACATGTTCTGCCACAAACTGGAGAAAATTATCGGTGAAATT**TAATGAAGAGCGACCAGAGACC
CAACACTG**

cpsH

AAGGGGTTGGTCTCATGTGGCTCTTCGATGCGTAAATACTTAGATTTGGACTACTCTCTGTTTTATGCCCTGTGG
GTGCTGATTTTAGTGCCGAACCAATGGTACCAGTTTCTTATCATTACCATCATCGTTTTGTTGCTTTTATGGAAGTC
TGAATTCCGTATTTCTATTAGTAACTCTTCTATTCTGTTTTTGTTATGGTTGTTCATCTATCTGTTCGCTATCTTAATTC
GCGGTACCCAGGAGGACATCACGTTCCAGCGTTTCATTGCGGAGTTACTGAAACTTATCTCAACTGGTTACGCCC
TTTTCTTTTACAACTATTACCGTAAAGCTGATTTTAATTCCTCAGTTGTGCGTAACGTGGTCAAAGTCAATTACTTC
GTTCTGTTCCTTATCACCGTTCTTTATCTTTTTTTCCCTATGCTTAAACCAACGTTGTTTGGACGCGAATTATTTAGT
ATTGAGTGGTTTCCTCACATGCGTATCCGTCTGGCCGCGTACTTCGAGTACGCCACATTAATCGGCCAGTTTATCC
TGTTTTCATATCCGATCCTGTTTCTGAAACCACAGAAGCGTATGGAGAATATTCTGATTTCGCTGCTTTTAACCATC
TGCAGTTATTTCTCTGGAGCCCGTATTTTATTGGTCTGTATGCTTGTACTGTTGGCCTCACTGTTGCTTGACTATATC
CTGTTTAAGACAAACCTGAAGTTAACTAAGAAAAACACGTTTATCCTGGGAATGACATTCCTTTTCATTACCGCGT
GCTTCTCTTATAACATCTGGTCTATTATTGAAAAAATTATTATGTACCGCAATCAGAGTACAATTACGCGTATGATTG
TTTACCAGGAGTCGATCATTGAGGTTCTGAAAGGGAACATCCTGTTTGGTCAAGGTATTCGCATTCCTTCCAGTG
AAGGTATCTTTTTGGGAAGCCATAGCACCTATATTAGCGTGTTCTACCGTACATCATTGCTGGGTATTGTCTTATATT
TCTCGGCTTTCATTTTGTTATATAAAGAAGCAATTAGTAAGAACTACAAAATCTACCGCTTATTTTTCTATACTTTGC
TTTGCTATACACTGTTTGAGGAGATCGACCCAAACCACTGGTCCATCGTGTTGCTGTTTTCGACATTCGGAATTGT
TGGGCGCGCAAAAAAG**TAATGAAGAGCGACCAGAGACCCAACACTG**

cpsI

AAGGGGTTGGTCTCATGTGGCTCTTCGATGAAGGAGAAGGTTACTGTGATCATTCCAATTTACAACAGTGAAG
CGTATTTAAAGGAATGTGTGCAGAGTGTCCTGCAGCAAACTCATCCGTTAATCGAGGTGATTTTGATTGACGACG
GATCTACTGATAACTCAGGAGGTATTTGCGATAATTTGAGCCAGGAAGATAACCGCATTTTAGTTTTTCATAAGAA
GAACGGAGGTGTGAGTAGCGCACGTAACCTTGGATTAGATAAAAGTACAGGTGAGTTCATTACTTTTGTTGACT
CTGATGATTTTGTCGCCCCAAATATGATTGAAATCATGTTAAAAAACCTGATCACAGAGAATGCGGACATCGCTGA
AGTAGACTTTGATATTAGTAACGAACGCGATTACCGCAAAAAGAAACGCCGTAATTTCTACAAGGTGTTTAAAAA
CAATAATTCTTTAAAGGAGTTTTTGTCGGGAAATCGCGTGGAAAATATCGTATGCACCAAACTTTACAAGAAAAG
TATTATCGGGAACTTACGTTTTGATGAGAACCTTAAAATTGGAGAGGACTTACTTTTTAACTGTAAGTTGTTATGT
CAGGAACATCGCATTGTTGTAGATACTACGTCTAGTTTATACACATACCGCATTGTTAAGACGTCTGCCATGAATCA
AAAGTTTAATGAGAACTCATTGGACTTTATCACAATCTTTAATGAAGTGTCGTCGCTTGTGCCGGCGAAGTTGGC
TAACTATGTCGAAGCCAAGTTCTTACGTGAGAAGATTAAATGTTTACGTAAGATGTTTGAGCTGGGGAGTAATATT
GATAACAAAATCAAGGTTCAACGCGAGATTTTTTTCAAAGACATCAAATCATATCCCTTCTACAAAGCCGTCAAAT
ATTTGTCATTGAAGGGTTTGCTGTCGTTCTACTTAATGAAATGCTCTCCGAAGCTTTATGTGATGGCCTATCGTCGT
TTCCAAAAGCAA**TAATGAAGAGCGACCAGAGACCCAACACTG**

cpsJ

AAGGGGTTGGTCTCATGTGGCTCTTCGATGATTAAGAAGATCGAAAAGGACTTAATTTCCGTCATTGTACCCATT
TATAACGTCGAGGATTATCTGGTGGAGTGCATTGAGTCCCTTATTGTACAGACTTACCGTAACATCGAAATTTTACT
TATTAATGATGGGAGTACAGACAATTGTGCCACGATTGCGAAAGAGTTCAGCGAACGTGATTGTCGCGTAATTTA
TATCGAAAAGTCCAACGGCGGATTATCGGAGGCACGTAATTACGGAATCTATCATTCTAAAGGCAAGTACCTTAC
ATTCGTCGACAGCGATGACAAGGTGAGTAGCGACTACATCGCCAACCTTTACAATGCCATTCAAAAACACGACA
GTAGTATTGCGATCGGCGGGTATCTTGAATTTTACGAACGCCATAACAGCATCCGCAATTACGAGTACCTTGACAA
GGTTATCCCAGTCGAAGAGGCTTTGTTGAATATGTATGACATCAAAACTTACGGAAGCATCTTCATCACAGCGTG
GGGCAAGCTGTTTCACAAGAGCATCTTCAATGATTTGGAATTTGCATTGAACAAGTACCATGAGGATGAGTTCTT
TAATTACAAGGCTTACCTTAAGGCCAATTCCATCACATACATCGACAAGCCATTGTACCACTACCGTATCCGTGTGG

GTTCTATTATGAACAACAGCGATAATGTAATTATTGCACGCAAGAAGTTAGACGTGCTTTCTGCTCTTGATGAACG
CATTAAGTTGATCACCAGCTTACGTAAGTATAGTGTTTTTCTTCAAAAAACAGAGATTTTCTATGTCAATCAGTACT
TCCGTACTAAAAAATTCCTGAAGCAGCAAAGCGTAATTTTCAAAGAGGACAATTACATTGACGCATATCGCATGT
ATGGGCGcCTTCTGCGTAAAGTCAAATTAGTCGACAAACTGAAATTAATCAAGAACCGTTTCTTC**TAATGAAGAG
CGACCAGAGACCCAACACTG**

cpsK

AAGGGGTTGGTCTCATGTGGCTCTTCGATGACTAATTTTGTATGTCATACACTTTACCACCTTTTGATTACAATCA
TTAAGTTGAAAGACAAGGAGAACACCCGCATTTTTATTTGCGATACCATCACGGATTATGAGACGTGGGTAAAG
ATCTTAAATGATCAAGGTATCCGTACAGAATCGTTTAAAGAGTTTGCGTATCGTGAGCAGCTTCAGAATAAAAATA
TTGAAGAAGTAATGGAATTAGTAGATAGCGATTTGAACCATTACTTCGAGCGCGTAGATACCCAGGTTTACCTTTT
TAACGACGACACTTTAATCGGACGTTACATGGTTTACCTTGGCAAGAACTACCACCTGATCGAAGATGGGTATAA
CTGCTTCCAGGCCAAGCTTTTTCTTGGAGGGTCTGTGGTCAAGCGTGTAATCAAGACATACTTGTTCAAGAAATA
CGTTCCATACGGTTTTTCTAAGTACTGCTTATCTATCGAAGTAAACTCCCTTGTGGGTCTGCCGCATGATATTCGTA
GTAAGAAATACAAGGAACTGCCACGTAAAAAATTGTTCGACTCGCTTAATAAAGAACAGAAAAGTCTTATCTTCA
AGATTTTCAAAACAAAACCATTGACTATCACCCCAAAGAGTGTATTGCTTTTGACACAACCGCTGGCACAGGATA
AGTGGTACAAAACGCCCACAGAACGTTTCCAGAGTATCCAAGAACAATATGATTACTTTGATGACATCGTCCAGG
AATATCGCACCTTAGGATACAACGTATATCTGAAGGTTCATCCACGCGATGTGGTCGACTATTCAAAGTTGCCGGT
GGAATTGCTTCCCTCCAACGTGCCAATGGAGATCATTGAATTAATGAGCACTGGACGTTTCGAGTGTGGTATCAC
CCATAGCTCTACCGCTTTAGATTCCTTAACGTGCGTTGATAAAAAGATCACTCTGGTCGATCTGAAGGATATCAAA
TAATGAAGAGCGACCAGAGACCCAACACTG

cpsL

AAGGGGTTGGTCTCATGTGGCTCTTCGATGAATACAAAGAAGTTGCTTCAATCGGGCTTCATCTATACATTAGGT
AACTTACTTGTCCAGGGATTAGCGTTCATCACTCTTCCTATTTATACACGCGTGATTTCTACAGAGGTTTACGGTCA
ATACTCCTTGTACGTGGCATGGATGAATATTATCATGTTGTTTATTGGGCTTCAAACCAGCGGTTCCCTTAGCTCCG
CCCGCGTAAAGTATGGGGAAGAATTTAAGTCATATTCAGGATCCGCTTTCTCAGTAGGCAATATTTGGTTTCTTAT
TATTTTATTAATTGCATTTTTGTTCCGTTCGTTTTTAGCACCTTTAGTAGGTTTCAGCGAGAGTATTTTTCTGCTTAT
GGTGTGTCAGTCCTATGCCAGTTATGTCGTAACCTTCTTTGGTCAATACTTCATCCAACAGCAACGCTCCCTTGCG
AACCTGATTTTATCCCTGGCGAACGCGGTATCTTCAGTGGCTTTGTCTTTGTTCTTAATCTTTCATTGGTCCGACGA
CTTTCTGAGTCGTGTATTCGGCGCTTTCGTTCCTACTATCATCACAGGTATTGTTGCTTTTGCATACATTTACTATCA
TAGTAAAAGTTTTTACAACCCAAAATACTTTCGTTTTATTGTAACCGTGTCGGTTCCTCTTATTTTCCACCTGCTGG
GCCACCAGCTGCTTGGGCAGCTTGATCGCATCATGTTGGCACGTCTTTATAACACAAAGGAGGTTGCGATGTATA
GTTTTGGCTATTCGCTTGGAATGATTATTCAAATTGTTCTTAACTCTATTAATATGGCCTGGATCCCATGGTTCTTCG
ATGCTCGTAAAGAGAAGCTGTTGCAGCTTAGCACTTATATTTCACGTTATTTATATTTGGGGGTTTTCCTTACTCTT
GGGTACTTGACAGTTTTCCCTGAATTGGCACAAATCATGGGGGGCGACAAGTATAGTTCCTCGGTACAATTCATT
TCGCTGATTATCGTGTCGTACTTCCTTGTATTTCTTTATACGTTCCCGGTAAATATCCAATTTTCTACGCAAACACTA
CCTGGATCCCAATCGGGACTTTGCTGGCCGCGGGTGTTAACTGGTTACTTAATTTGGTCCTGATTCCGCACTATGC
TGCGTATGGGGCAGCAATGGCAACGATTATTAGCTATCTGGCATTACTTATTTTTCATCACATCGTTAGTAAAGTAA
AGTACCACTACAGCGATGTGTCGGTACGTCAATACATCATCTTATCTGGGATTGTGTTTAGTTACGCGATGTTAATG
AATATGTTCTTAGACAACATCGTCATCCGTTGGTCCCTGGGCATCATCATTCTGATCGTATACAGTATCGTATTCCAG
AAGGTAATTCTGGACCTGCTGTCGAAAAAGCGCCGTCGTCGC**TAATGAAGAGCGACCAGAGACCCAACACTG**

neuB

AAGGGGTTGGTCTCATGTGGCTCTTCGATGGTATATATCATTGCCGAGATTGGGTGCAATCACAATGGTGACATT
AACCTTGCTAAAAAGATGGTGGACGTTGCCGTAAGTTGTGGTGTAGATGCTGTTAAGTTCCAGACTTTCAAGGC
AGAAAAACTTATCTCTAAGTTTGCCCCAAAGGCCGAGTATCAAAAAGAGACAACCGGCACGGCTGATAGTCAGC

TGGAAATGACAAAGCGTTTGGAATTATCATTCGAAGAATATTTGGAAATGCGCGATTACGCCATCTCAAAGGGAG
TGGAGACGTTCTCAACTCCGTTTGACGAAGAGAGTTTAGAATTCCTGATTTCAACAGATATGCCTATCTATAAGAT
TCCTAGCGGCGAGATTACTAATCTGCCATATTTAGAAAAGATCGGTAAGCAACAAAAAAAGGTCATTTTGAGTAC
CGGTATGGCCGTAATGGAGGAAATTCATCAGGCGGTTAATATCCTGCGTCAGAATGGAACTACAGACATTTCTATC
CTTCATTGCACAACTGAATACCCTACTCCGTATCCTTCACTTAACTTAAACGTAATCCATACGTTGAAGGATGAGTT
CAAAGACCTTACCATCGGCTACTCCGATCACAGTATTGGTTCTGAGGTGCCGATCGCAGCAGCAGCAATGGGTG
CTGAGGTCATTGAAAAACATTTTACCCTTGATACTAATATGGAAGGCCCAGATCACAAGGCAAGCGCCACACCCG
ATATTCTTGCCGCGCTGGTCAAGGGTGTTCGCATCGTTGAGCAAGCCCTGGGCCGCTTCGAGAAAATCCCAGAC
CCCGTGGAGGAGAAGAATAAAATCGTTGCGCGTAAAAGCGTAGTAGCCCTTAAACCCATTAAGAAAGGGGACA
TTTATTCCATTGAAAACATCACTGTTAAGCGCCCCGGGAACGGAATCTCCCCCATGAACTGGTATGATATTTTAGG
CCAGGAAGCTCAGGACGACTTTGAGGAGGATGAGGTGATCCGCGATTCCCGCTTTGAGAACCAACTTCCAGAA
CTG**TAATGAAGAGCGACCAGAGACCCAACACTG**

neuC

AAGGGGTTGGTCTCATGTGGCTCTTCGATGAAAAAGATCTGCTTGGTGACAGGTTCTCGTGCTGAGTACGGAA
TCATGAAACCATTGATCCAACGTCTGTCAAAGGATAAAGAAGTGAACCTGCAAATCATTGCAACTGCTATGCATTT
GGAGGAGAAGTACGGTTACACGTACCGCCAGATCGAGGAGGATGGTTTTGACATTGCTTACAAGGTCCCTTTGC
ACCTGTACGATACCGATCGCCGCACCGTGAGCACTGCCATGGCTCACTTGCAGCTGGGGTTGACGAAAATTTTC
GATAAGGAAGATTACGACTTAGTCATTATTTTAGGGGACCGTTACGAAATGTTGCCAGTGGTCAATGTTGCACTG
ATTTACAACGTTCCCGTTTGTCACTTGCACGGTGGGGAGACGTCATTGGGCAACTTTGACGAGTACATTCGCCAC
GCCGTAACTAAAATGTCGCATCTTCACTTGGTGTCCACCGAAGATTTCCGCCAGCGCGTCATTCAAATGGGCGAA
CAACCACAGTTCGTGATTAACACAGGGGCATTAGGCGTTGAAAACGCTCTGTCTATCCCTTCTCTGACGAAAGAA
GCTATTGAAAAGCAGTTGGGAATTGTCTTGGAGGAATCCTACTTTGTCGTGTTATATCATCCTGTCACCTTCGAGC
AGGGGAAGTCTGCAGGAGAACAGATGAAAGCTGTGCTGTCGGCTCTTTCTAAGTTCGGGGTCCAGTGCCTGTT
CATCGGGTCCAACTCTGATACCGGAAGCGATGACATCGCAAAGGCGATTAACACATATTTAATCAATCACGAAAA
TTCATACTGCTTCGCGTCCCTTTCAACCCAGCTTTATCACAGCTTAATCCGCCATAGCCTGGGGCTGATCGGAAAC
AGTTCGTCTGGACTTATCGAGGTCCCATCGCTGATGAAGCCCACGCTTAACATCGGGGATCGTCAGAAGGGCCG
TTTACACGGGGAATCTGTTGTGTCTGTTCCAGTAGAAACATCGTCGGTACTTGAAGGACTGTCCAAGCTGAATGA
AGTTACGAATTTTGACAACCCTTACTATAAGGGAAATGCCTCTTCCATCGCTTATGAAGCAATCAAGTTATACTTAA
AAGACGAACCCTCTATCTCCCAGCCCTTTTACGATTTAAAGGAAAACAACTTGAAG**TAATGAAGAGCGACCAG
AGACCCAACACTG**

neuA

AAGGGGTTGGTCTCATGTGGCTCTTCGATGAAACCTATTTGTATCATTCCTGCACGTTCAGGATCGAAAGGATTA
CCTGACAAAAACATGTTATTTCTGGCCGGCAAGCCTATGATTTTTCACACTATTGACGCCGCGATTGAGAGCGGA
ATGTTCGACAAAAAGGATATCTTCGTGAGTACAGATTCAGAGTTGTACCGCGAGATCTGTCTTGAGCGCGGTATC
AGCGTAGTCATGCGCAAACCGGAATTGTCGACTGACCAAGCAACCTCTTACGATATGCTGAAAGATTTTCTTTCA
GACTATGAAGATAATCAGGAATTTGTCCTTCTGCAGGTCACAAGCCCCTTACGTAAGAGCTGGCATATTAAAGAA
GCCATGGAGTATTATTCTTCTCACGACGTTGATAACGTAGTATCCTTTTCAGAAGTAGAAAAGCACCCAGGATTAT
TTACGACGCTTAGCGATAAGGGATATGCCATCGACATGGTCGGGGCCGATAAGGGTTACCGTCGCCAAGACCTT
CAGCCTTTGTATTATCCTAATGGGGCGATTTTTATTAGCAATAAAGAGACTTACTTGCGTGAAAAAAGTTTCTTCA
CGTCCCGCACATACGCATATCAAATGGCGAAAGAATTTTCTCTTGATGTAGACACGCGCGATGATTTTATTCATGT
GATCGGTCATTTATTCTTCGACTACGCGATTCGTGAAAAAGAAAACAAGGTGTTCTACAAGGAAGGCTACTCTCG
TTTGTTTAACCGTGAAGCATCCAAGATTATTCTGGGAGATTCAAAAACCATCTCTATTAGTTTGGAAAATTATCATA
ACTACTCACAAGGGGGAGTTACGCTTGCTACTATGCTTGAAAATTTGCCTAACTTTCTTACCGCAAACGTCACGG
AGGCCTTTGTGTCCATTGGCGTAAACGACCTTATCACCGGATATTCTGTTGAGGAGATCTTCTCTAATTTCCAAAA
ATTATACAGTCTGTTAGCAGAGAACAAAATTAAAATGCGTTTCACTACTATTGCGTACACCCTGTTTCGTGAAACA
GTGAATAACGCAGACATCGAGAAAATCAACCAGTGGTTAACGGAATTCTGCTATCAGAATCAGATCCCGCTGCTG

GATATCAATCGTTTTCTTTCTAAGGATGGCAATTTGAACTATCACTTAACTTCGGATGGCTTACATTTCACGCAGGAAGCGAATGACTTGTTGCAAAGCCAGTATCAGCTTTTCGTCGATGAGGTCAAAACCTTA**TAATGAAGAGCGACCAGAGACCCAACACTG**

GBS-IV

cpsG

AAGGGGTTGGTCTCATGTGGCTCTTCGATGATTTTTGTAACAGTGGGAACCCATGAGCAACAGTTCAACCGTCTGATTAAGGAAGTAGACCGCCTGAAGGGGACCGACGCAATTGACCAAGAAGTATTCATCCAGACGGGTTATTCCGACTTTGAACCACAAAACTGTCAATGGTCGAAGTTTCTGTCATATGACGATATGAACTCTTATATGAAAGAGGCTGAAATTGTCATCACTCACGGGGGGCCTGCGACGTTCATGAATGCCGTTTCCAAGGGGAAAAAAACCATCGTTGTGCCACGTCAGGAACAGTTCGGCGAGCACGTCAACAATCATCAGGTTGACTTCGTAAACAAAGTTAAAACCATGTATAACTTTGATATTGTTGTGGACATTGAACGCTTACAAAATGTAGTTTATGAGGGAACAATGAACCGTCCATTTTTGGAGACGAACCGCTCTAATTTTATTGAGGAGTTCAAGGTCATTTTGAAAGAGTTGTGCGATGAAAATCAA**TAATGAAGAGCGACCAGAGACCCAACACTG**

cpsH

AAGGGGTTGGTCTCATGTGGCTCTTCGATGAAAATTAATAAAAATTCACTTTTTTATATCGCAATCTTCCTGGTCAACTTCTTCAAGTCGTTGGGGCTTGGTGAAGGTAACAGCACTTACAAGATTGTTATGTTCGTCGCAATCTTTTTATGCGGCATTAAATTCTTGCTTGATTCGCTTTATTTCGAGCGCCGTAAACTGGTGATTATTTTCCTTTTGTTCATCGCAACCATCTTAAATTTGTTCTTTGTTCATAAAGTCACATTCATCTTAACGCTTATCTTCTTCTTGGCCTTGAAGGATATTTCCCTTAAAAAAGCCTTCTCGATTATTATTGGATCACGCATTTTGGGCGTACTGTTGAACCAGATTTTCGTCAAACTTGATCTTATTGAGATTAAGTACATTAACTTTTATCGTGACGGGCAATTCATTCTGCGTTCAGACTTAGGTTTCGGACATCCCAACTTTATCCATAATTTTTTCGCTGTGACAGTATTTCTGTACGTAACCTTATTTTACCGCAAGTTGCGTTTGATCACAATCGCCTTCATCTTAACCTTGAATTATTTTTTATATCAGTACACGTATTCACGTACTGGGTACTACATCGTGCTTTTGTTCATTCTTATCATTTACGTTACCAAGAACAACTTGATCCGTAAGATTTTTATGATCGTTGCTCCTTACATCCAGCTTTTTTTGCTGGCGTTCACTTTCCTTTGCTCGACAATCTTCTTTAATTCCAACTTTGTGCAAAAATTGGATTCACTGCTTACGGGACGTTTAAACTATGCCCACTTGCAACTTGTCGACGGTCTTACTTTGTTCGGTAATTCCTTTAAGGAAACGTCTGTACTGTTCGACAACTCTTACAGTATGCTGCTGTCCATGTACGGTGTGGTCCTGACAATGTTTTGTATGATCATTTACTATATCTATTCCAAAAAGGTGAATGTTGTGGAGCTGCAGATTTTGCTGTTTATCATGAGTATCGTCCTGTTCACTGAGAGCTTCTATCCGAGCATTGTTATGAATATTAGTTGGATGGTATTCGGGAAGATTTTTTGTGGGGGGGTAGATGACTTGCAGCGTGAGTTCACTTGGACCGCCAATAAAAAT**TAATGAAGAGCGACCAGAGACCCAACACTG**

cpsI

AAGGGGTTGGTCTCATGTGGCTCTTCGATGATTGAAAAAACGATGATCCCTAAGAAGATTCACTATTGCTGGTTCGGTGGAAATCCGAAGAGTGAACTTTTGCTTAAATGCATCAAAAGCTGGGAAAAGTACTGCCCCGACTACGAGATCATTGAGTGGAACGAACAAAACTACGACGTAAATAAGATTACTTATACACGCCAAGCATATAAAGAGGGTAAGTACGCCTTCGTCTCAGACTATGCACGTCTGGATATCATCTACCAATACGGGGGTATCTACTTGGATACAGATGTGGAATTGATTAAACCTATTGACGACCTTTTAAATTGCGAGTCGTACTTTGCTTGTGAATTACCCGGCGAGGTTAATACTGGCTTGGGATTCGGCTCGATTTCACGCAACCCTTTTATCAAGGAGAACATGAAAATCTATGAAGATACCGAATACTATTCCCTTAAAAAAACATGTGTTGCTATTACCACAGACTTATTGACAGAACAGGGACTTAAATATATTAATAAGATTCAGGTCATTAATGGGATCTTAATTTACCCTCCAAGCTACTTTTGTCCCGTCAACATGCTGACGAACAAAATTAATAT

CAAGGAAGAAACCTTGACGATTCATCACTATGGCCGCAACTGGGGAGGTGAGAAAAAAATTATTGATTTAGGTC
GTATCAAGCTTGGGGTTCACCTTTTGCTGGACAAGATTTTCGGGTATGGAACTTACCGTAATATTCTGAAAGCCG
TAAAAAAG**TAATGAAGAGCGACCAGAGACCCAACACTG**

cpsJ

AAGGGGTTGGTCTCATGTGGCTCTTCGATGGAAAATATTCTGATCTCTATCATTATTCCTATTTACAACGTGGAAA
AATTCCTGGAGGAATGTATTGACAGCGTACTTAATCAGACCTACAAAAATATTGAAATTCTTTTAGTTGATGATGG
ATCCACTGATAACTCGGGAATTATCTGCGATAACTATAGTCTGAAGGATAAACGCATTCGTGTTTTTCACAAAAAC
AATGGCGGCTTGTCCGATGCGCGTAATTTCGGAGTGGTGAATGCCGAGGGGCGCTATATCTCTTTTATTGATAGT
GATGATTACATTGATAAGAATTATATCCGTAAAATGTACTATTGTCTGAGTAATCATAAAGTGGACATGGTCATTTGT
AATTATCTGAGTGTGTATAATAACGACTTAAAACCGATTGTATCTCGTTTACAAGACTGTATCATTTATTCCCGCGTT
GAGTTATTTTCAAACTTATATGGTAAGTACAAAGATCCATTTACGACAGCCTGGGGGTCATTGATTCGCACCGACA
TCGCTAAAAAGGTAACATTTCCTATCGGTAAATTGCATGAGGACGAGTTCACAACCTACAAATATTACTTATACAG
TGACAAGATCGCTTTTGTGCCTGAGCCATTGTACTTCTATCGTCGCCGTGAGGCGTCTATTATGAGTAGCAGTTAC
AGTAAACGCAACTTAGATTCCCTTGAAGCGTATGAGGAGCGCATTAAAATTTTAGAAAAGAACAATATCAGTATT
TCAGAGACTGTCTATATTTACTTATCATTGATCCTTTATCATATTTACCGTATCCGCAAGTATCCTGAGGTTTGTAATA
AACAAGAGCTGCTTGACAAGTTCAATAATTATTATCTTAAATATAAAAACATTTTAGGTCTGAAGAACCGCTTGAA
GATTATCACCCTGAAGAACCTTGGAATCCCGTACCAGCGTTATAAACATAAACTT**TAATGAAGAGCGACCAGAG
ACCCAACACTG**

cpsK

AAGGGGTTGGTCTCATGTGGCTCTTCGATGAAGAAGATCTACATCTGTCATACAGTCTACCATTTGTTAATTACAA
TTATCAAGATCTCTATTATGCGTGAACAACGTCGTTCAATTATTATCCTGGCTAATAAGATTGTAAACATTGATGACC
TGTGCAAACGTACCAACAAAGAAGGAATCTTATGTTTCAAGTTAGATGAAACCAGCTCGACACCAACGACGGTG
GACACAATCACTATTGACTTAATTATTGAAACGAACGGTACAATCTACCTGTTCAACGATGATACCAGTGTGGCGC
GTCAGATTGTACGTGCCCAAAAAAATTATCATTTAATCGAAGATGGCTATAATTGTTTTCAGGCTAAATTATTTCTT
GGCGGCAGCGTAGTCAAACGTGTCATCAAAACCTACTTATTCAAGAAATACGTTCCTTATGGTTTTTCAAAATACT
GCCTGTCTATTGAGGTAAATTCACTGGTTGGACTGCCCCACGACATCCGCTCGAAGAAATACAAAGAGTTGCCCC
GCAAGAAACTTTTTGACTCTCTTAACAAAGAACAAAAATCGTTAATTTTTAAAATCTTCAAAACAAAACCACTTAC
GATTACACCCAAATCCGTCCTGCTGTTAACCCAACCTTTGGCACAAGATAAGTGTTACAAGACTCCGACCGAGCG
TTTTCAATCTATCCAGGAACAGTATGACTACTTCGACGACATTGTCCAAGAATACCGCACTTTGGGATATAACGTG
TATTTGAAAGTGCACCCGCGTGATGTCGTAGACTATAGCAAATTGCGCGTTGAGCTGCTTCCGTCTAACATTCCGA
TGGAAATCATTGAGTTAATGTTGACAGGGCGTTTTGAATGCGGAATTACACATTCATCTACCGCGTTAGACTTTTT
AACTTGCGTGGACAAGAAAATCACATTAGTCGATTTGAAGGATATCAAA**TAATGAAGAGCGACCAGAGACCCA
ACACTG**

cpsM

AAGGGGTTGGTCTCATGTGGCTCTTCGATGATTTACAATGAAAGTTCACTGGGTCGTCAAATTAAAATTAGCGTG
ATCGTCCCTGTGTATAATAGCAAGCAGTATTTGATCGCATGCGTGGATTCAATTCGCAAACAAACCTATAAGAACC
TGGAAATTATCCTTGTCAATGATGGAAGTACTGATGGTTCCAAAGAACTGTGCGAGGAAATCCGTAAATCTGACG
AGCGTATTAAGACGTTTCATAAGACTAACGGAGGGCAATCGAGTGCACGCAACCTTGGAATCCTGTACAGCACG
GGGGACCTGATCGGATTTGTAGATTCCGACGACACTATCGATCCGAAAATGTACGAGACGCTGCTGAACATTTAT
GAAGATGAACAGGTAGACTGGGTGCAGTGTAATCATAAAAAAATCTACAGTAACGGTGTAAATTTGTACTACAAC
GGCCCAGAGTACTATAATGTTTTGAACAAACAAGACTTTCTTTATGAATTCTTATCAACGAATAAGATTTTCTCTTC
TGTATGTGAGGGACTTCTTTCTCGTGACCTGGCCTTGAAAATTAAATTCCGTGAAGAAAAAAAATATGAGGACAC
GCAGTTTTATTTCGATCTGATTAAGAACGCAAATAAATTCGTGATCATCTCACAGCCGTTCTACAATTATTACTATCG
TAAAAACAGTACGACAACAAGCTCGTACTCATCATATCAATGGGACATCATTGATATCTGTACGGAATGCTATTACT

ACGCTAAGGATTTTAATGGTTTTGAGGAGGTGGCCTTCTCTCGTCTTTTTGGTGCATACAGCTTGGTTGCTAACA AGATTGTGTACAACAAAGATTATCGCAAAACAGAAGAGTTAAGA**TAATGAAGAGCGACCAGAGACCCAACACT G**

<u>GBS-V</u>

cpsI

AAGGGGTTGGTCTCATGTGGCTCTTCGATGTTATACATCTTCACCCCAACATTTAATCGCGGATACCGCCTGTCGT ACTTGTACGACTCTTTGTGTAACCAGACCAACAAAAACTTTATTTGGTTAATTGTGGACGACGGATCAGAAGATA GCACAAAAGAGATTGTGTCAAATTACATTAAGGAGAATAAAGTCTCTATTGTTTACTTGTATAAACGCAATGGAG GCAAACATTCAGCCTATAACTTGGCAATGCGTTACATGCAGCCCAGCGATTACCACGTCTGTGTTGATTCAGACG ATTGGCTTTTGGAGGACGCAGTCGAAATTATCTTTAAGGATCTTGAGTCCTTAACGCTGTCGAATCGCTACGTCG GCTTGGTTTACCCACGTTATTCTTTAAATCAGGGTAACAATTGGCTTAACCCTAAAATTTTGGAGGTCAATATTCCC GATCTTAAATACAAGTATCATCTGAAAATTGAAACTTGTATTGTCATCAACAACGCTTATCTTGTTGATTTTGAATTT CCGTGTTTCGAAGGGGAGAATTTCTTGTCAGAAGAGATTATGTATATTTACTTGTCTAAGAAGGGATATTTTTGTC CGCAGAATCGCAAGATTTATTGTTTTGACTATCTGGAAGATGGATTGACAAGTAACATTTTTAAACTTTGGCGTAA GAATTTTAAAGGTACGATTTTCAGTCTTGAAAATAGTTATATGTATGTAATGAGCTTCCCCAATATTTTTGACCGCT GGTGGTCCGCCATCAAAATCAAAATGAATATCCAGGCGTTGAAAATGACGACGCTGGGGGTTATCCCTACATTAA AATCCGAAGAGGCCGGTTGGAAAATTTTACTGGGGTTTTCTTATCTGTGGAAAGTCGCCCGTTTCAAAAAAAGT GAG**TAATGAAGAGCGACCAGAGACCCAACACTG**

cpsJ

AAGGGGTTGGTCTCATGTGGCTCTTCGATGGATAAAGTGAGTATCATCATTCCTGTCTATAACGTGCAGTCCTTTC TTAACGAATGCATCGAGTCGGTCTTGGCACAGACATACAGCAACCTTGAGATCATCCTGGTCAATGACGGCTCGA CTGACAATAGCGGAGATATTTGTGATTATTACTCAGAGATCGATGGTCGTATCTTCGTATTTCACAAAAACAACGG AGGATTATCTGATGCGCGCAATTATGGGATCAGTCGCGCTACGGGGGATTATATCTACTTACTTGACTCGGACGAC TATTTGTATAAAGAAGATGCCATCGAGCGTATGGTAGAGTTTTCGGAGAAGTACAATTCTGAAATCGTCCTTGGAT GCTATGTCGAGAAGCGTGAACAACACATCATCAACATCGTATTAGAGGACGAGATGATTGAGACTATCTCACCAG TACAAGCTATTCAGAATATTTACAACTATGATGCATACCGCGCTATCTTCACCGTTGCACATAACAAACTGTACAAG CGTGAATTGTTTTCAACGCTGTGCTACCCCGTTGGGAAACTTCACGAGGATGAGTTCTTGACATACAAGTTGTAC TTGAAAGCTAAGAATATCATCTTCTTCCGCTATAACACGTACGCTTACCGTATTCGCGAAAACTCCATCATGACGG GATCATATAACATCAAGCGTTTGCACGCTGTGGAAGCTCTGAAAGAGCGCATTTACTTATTGGAAAAATACCCGG ATCTTGTATTCCAGTCTGAGCGTGCGCTGATTAAAACTATGGAAGTCAACTTGATCGAGCTGTACAAAAATAACTT CTATAAGGAGTTCCACACGCTGAAAACGGAGTATAAAAAAACCATTTTTGACTTCATCAAAAAGCAGCGCATGTT GTTAAAAATTAAGTACTACTTGAAATATTGCGTAGTTCATTTCAAAATTTTGAATTGCAAGCGTAAAAAGAAGATC AACAAA**TAATGAAGAGCGACCAGAGACCCAACACTG**

cpsM

AAGGGGTTGGTCTCATGTGGCTCTTCGATGATCCCAAAAGTGATTCACTACTGTTGGTTCGGCGGTAATCCTCTG CCGGATAATCTGAAGAAATATATTAAAACCTGGCGTGAACAGTGTCCCGATTACGAAATTATTGAATGGAACGAA CACAATTATGACGTCAGTAAGAACGTCTTTATGCGCGAAGCGTACACTAAAAAGAACTTTGCATACGTCTCCGATT ACGCCCGCCTGGACATTATCTACACTTACGGGGGGTTTTACTTGGATACAGACGTAGAACTTTTAAAGTCCTTGG ACCCTCTGCGTATCCATGAATGCTTCCTTGCGCGCGAGATCTCATGCGATGTCAACACTGGGCTGATTATCGGCGC TGTAAAGGGACATCATTTCTTAAAATCTAATATGTCAATCTACGACAAGTCAGACTTAACCTCCCTGAATAAAACTT GTGTCGAAGTAACTACCAATTTACTGATCAACCGTGGATTGAAAAATAAGAATATCATTCAAAAGATCGACGATAT CACGATTTATCCCCGTAACTACTTCAATCCGAAAAATCTGTTAACAGGGAAGGTGGATTGTCTTACCAGCGTTACG

TATTCTATCCACCACTACGAAGGAAGCTGGAAATCAAGCTCTTTTATTTCCGATAGCTTGAAGATCCGCGTCCGTC
TTATCATCGATTTCCTGTTCGGATACGGGACGTACCGTATGTTACTTCGTTTTTTAAAATTGAAGAAA**TAATGAAG
AGCGACCAGAGACCCAACACTG**

cpsO

AAGGGGTTGGTCTCATGTGGCTCTTCGATGGAACTGATTAGTATCATTGTTCCAGTGTATAATGGGGAGATTTAC
ATCGGACGCTGTCTTGACAGCATCCTTGAACAGACGTACCAAAATCTGGAAATCATTATTATTGATGATGGAAGTT
CGGATCGTACAGGAGACATCTGTGAGAAGTATTTTTTGGAAGATCGCCGTATTAAATACTTTTACCAAGAGAACC
GTGGTCAATCCGTCGCCCGCAATAACGGAGTTCTGCGTTGCACGGGCGATTGGATCGCGTTCTTAGATAGCGAC
GATGTGTACTTGCCTTACTCGATTGAGGTAATGTATAACATTCAGAAAGCAACGAATGCTGATATTGTACTGACATC
TATCGGGAACTTTAACAACACTTATAACACGAGTATTAACTCCCAGTACCTGAAAGAGATCAAATTGTACACCCTG
GAAGTTGCGTTGGAAGAAATGTACTACGGAAAAACATATGGAGTCTCCCCCCTGGCGAAATTGTATCCCCGTTCA
AATTTGTTATCGAACCCTTATCCTGAAGGCAAGATTCATGAGGATATGGATACGACGTTCAAACTGATCAGCTGTG
CCTCGAAGATCGCAGTATGTGACATTGTCACGGCCGTCGTGTATTTCAGTGACAACTCCACTACTCGTACTAAGTT
TAATGAGCGTATGCTTTACTTTTTTGAAGCGATCCAAAATAACATTGTATTTATCAATTTAAATTTTCCGCACAACAC
CTCATTAATTTCCGCAGTAATTTATAATGAGGTATTCGGGGGAATTGATATTTGCGGCAAAATGATCGACTTCAAAT
TGTACGATACGGTCGACTATTATCGTAAAAAGTATCGCAAATATTTCAAAACTATTCTGTTCAACAACCGCATCAGC
GTAAAGGAAAAGGTCAAGTATATCCTGTTCATCAGCTCGATTCGTTACTTTACGATTGTACGCAAGATCTATAACTT
ACGTTTGTCGCGTAATGGA**TAATGAAGAGCGACCAGAGACCCAACACTG**

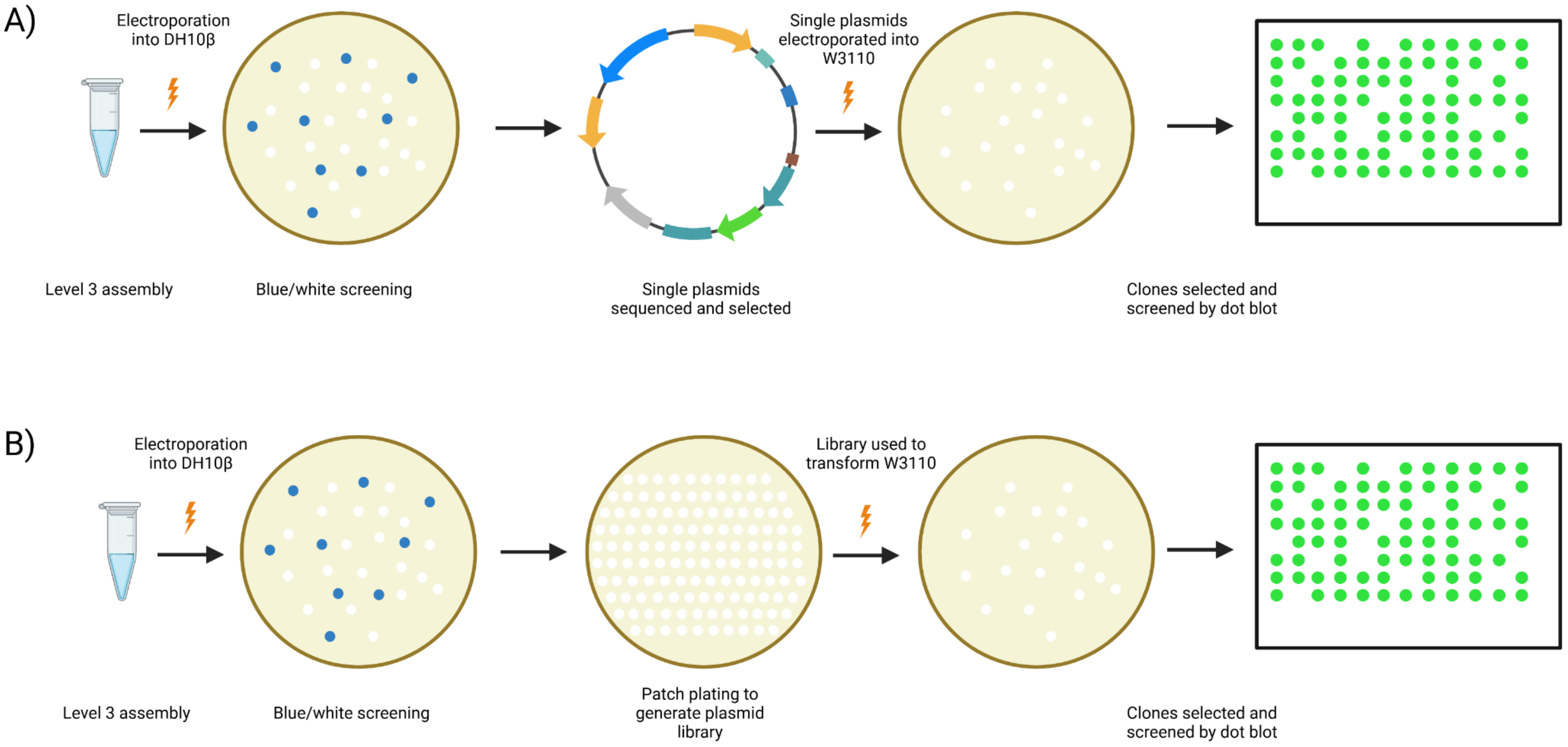

Supplementary figure S1. Two approaches taken for generation of plasmids encoding GBS-III *cps* locus. A) Approach one - following transformation of DH10β, plasmids were verified by sequencing and those that were correctly assembled were transformed into W3110. B) Approach two - following transformation of DH10β, colonies were patch plated and used to prepared a library of plasmids which was then used to transform W3110. W3110 transformants for both approaches were screened initially by dot blot. Figure created with Biorender.com

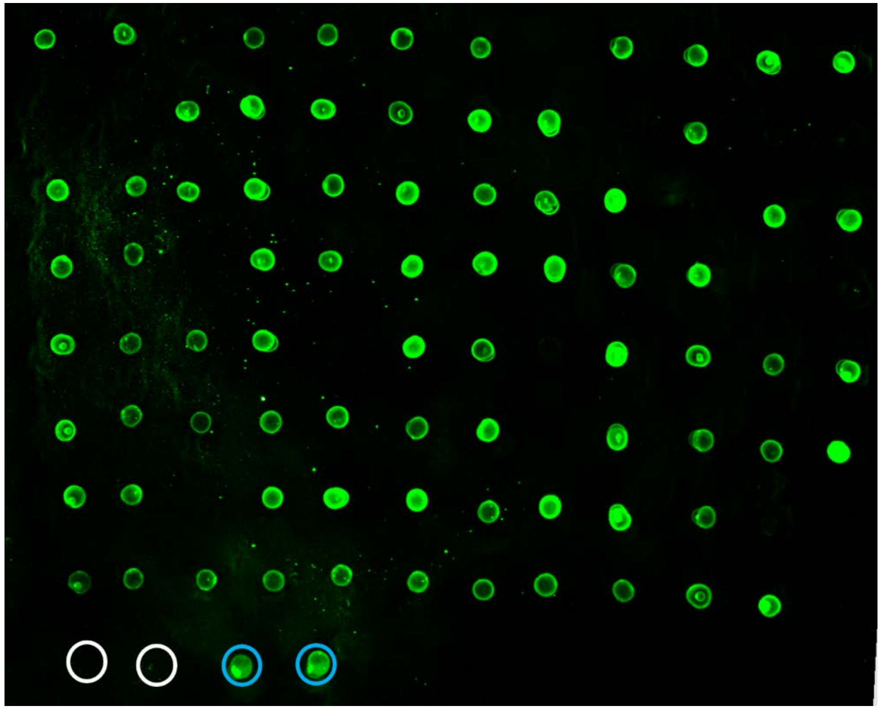

Supplementary figure S2. Immunoblot screening of W3110 transformed with an undefined library of level 3 plasmids encoding CPS-III glycan.

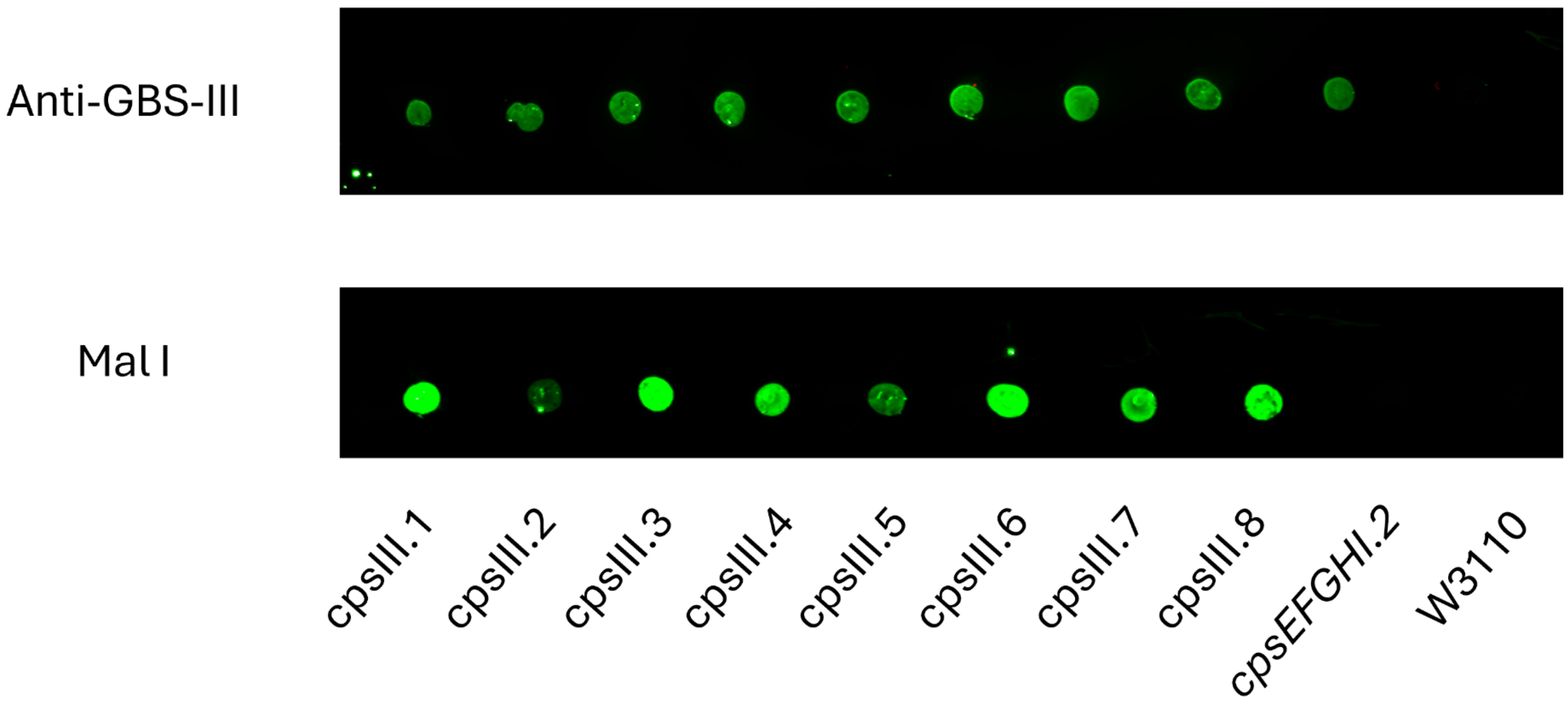

Supplementary figure S3. Detection of GBS-III *cps* expression via immunoblot with anti-GBS-III antisera and Mal I.
